# Warming increases Bacterial Panicle Blight (*Burkholderia glumae*) occurrences and impacts on USA rice production

**DOI:** 10.1371/journal.pone.0219199

**Published:** 2019-07-11

**Authors:** Aaron M. Shew, Alvaro Durand-Morat, Lawton L. Nalley, Xin-Gen Zhou, Clemencia Rojas, Greg Thoma

**Affiliations:** 1 College of Agriculture, Arkansas State University, Jonesboro, Arkansas, United States of America; 2 Cooperative Extension Service, University of Arkansas System Division of Agriculture, Little Rock, Arkansas, United States of America; 3 Department of Agricultural Economics and Agribusiness, University of Arkansas, Fayetteville, Arkansas, United States of America; 4 Texas A&M AgriLife Research Center, Beaumont, Texas, United States of America; 5 Department of Plant Pathology, University of Arkansas, Fayetteville, Arkansas, United States of America; 6 Department of Chemical Engineering, University of Arkansas, Fayetteville, Arkansas, United States of America; International Rice Research Institute, PHILIPPINES

## Abstract

Bacterial Panicle Blight (BPB), caused by *Burkholderia glumae*, is a bacterial disease in rice (*Oryza sativa*) that reduces rice yield and quality for producers and consequently creates higher market prices for consumers. BPB is caused by the simultaneous occurrence of high daily minimum temperatures (~22°C) and relative humidity (~77%), which may increase under the current scenario of global warming. This study hypothesized that the economic damage from warming may cause an increase in economic losses, though at a decreasing rate per degree. Thus, this study estimates the yield losses associated with BPB occurrences at the county level in the Mid-South United States (US) for annual rice production in 2003–2013 and under +1–3°C warming scenarios using daily weather information with appropriate thresholds. From the estimated losses, the total production potential of a BPB-resistant rice was quantified using a spatial equilibrium trade model to further estimate market welfare changes with the counterfactual scenario that all US county-level rice production were BPB resistant. Results from the study indicate that the alleviation of BPB would represent a $69 million USD increase in consumer surplus in the US and a concomitant increase in rice production that would feed an additional 1.46 million people annually assuming a global average consumption of 54 Kg per person. Under the 1°C warming scenario, BPB occurrences and production losses would cause price increases for rice and subsequently result in a $112 million USD annual decrease in consumer surplus in the US and a loss of production equivalent to feeding 2.17 million people. Under a 3°C warming scenario, production losses due to BPB cause an annual reduction of $204 million USD in consumer surplus in the US, and a loss in production sufficient to feed 3.98 million people a year. As global warming intensifies, BPB could become a more common and formidable rice disease to combat, and breeding for BPB resistance would be the primary line-of-defense as currently no effective chemical options are available. The results of this study inform agriculturalists, policymakers, and economists about the value of BPB-resistance in the international rice market and also help support efforts to focus future breeding toward climate change impact resilience.

## Introduction

Rice consumption accounts for more than half of the daily caloric intake of over three billion people globally, mostly in low-income countries [1]. Despite substantial rice consumption worldwide, 90% of the world’s rice supply is produced in only 15 countries, primarily in Asia [2]. Abiotic events such as drought and heat stress, and biotic events such as diseases and pests, can alter the global rice supply and cause subsequent food insecurity, as well as increase the environmental impacts of agricultural land use by reducing efficiency. Accordingly, this study focuses specifically on a rice disease called Bacterial Panicle Blight (BPB), caused by the bacterium *Burkholderia glumae*, in the United States of America (US). Supply shocks from BPB can have a substantial impact on the international rice market, even in relatively minor rice-producing countries such as the US. While the US generates only about 1.3% of world rice production annually [3], it exported rice to over 120 countries and accounted for 7.7% of the global rice trade in the years 2014–2016 [3]. Because the global rice market is so thinly traded, with only around 9% to 10% of production being traded internationally since 2014 [3], supply shocks in the US can have far-reaching implications for global food security [4]. Previous studies have quantified the supply shocks and food security implications of rice blast, caused by the fungus *Magnaporthe oryzae* and rice sheath blight, caused by the fungus *Rhizoctonia solani* AG1-1A, in the Mid-South rice-growing region of the US [5,6], but no studies have investigated these impacts for BPB. The BPB disease is currently a threat to rice production in many regions of the world, and with an increase in global temperatures, it is likely that this disease will become more prevalent and economically damaging throughout areas dependent on rice production [7]. Therefore, this study quantifies the occurrences and estimates the economic and environmental impacts of BPB in US rice production under current weather regimes and for +1°C, +2°C, and +3°C warming scenarios.

Unlike rice blast and sheath blight, the yield losses associated with BPB cannot be mitigated with applications of any pesticides currently used in rice production. Chemical methods to control this bacterial disease are not available currently in the US or any other country. BPB has the potential to reduce yield by up to 75% in severely infested fields as it causes several types of damage, including grain abortion, floret sterility, and milling quality reduction [8–13]. Significant yield losses from BPB were reported in the US Mid-South in 1996, 2000, 2010, and 2011 [14–18]. In the state of Louisiana, yield losses in 1995 and 1998 were estimated at 40% for severely infected fields [15,16]. The disease was so severe in the US Mid-South in 2010 that resulted in up to 50% yield loss in susceptible varieties across the region [14]. Additionally, the occurrences of BPB resulted in an estimated 10% to 20% yield loss in the Texas Rice Belt in 2010 [17]. Occurrences of the BPB disease are heavily dependent on weather conditions such as prolonged high daily minimum temperatures and frequent rainfall during the panicle emergence and flowering periods of rice, which raises relative humidity levels [19–21].

BPB was first identified in Japan in 1967 as the cause of grain rotting and seedling blight on rice and was called bacterial grain rot [22,23]. Since then, BPB has been reported in other Asian countries, Central America, Latin America, North America, and Africa [8,9,13,24–28]. In 1996–1997, the bacterial plant pathogen *B*. *glumae* (formerly *Pseudomonas glumae*) was identified as a cause of panicle blighting in the US Mid-South [16]. Currently, there are no commercially available rice cultivars with acceptable levels of resistance to BPB, although it is possible to breed for resistance by harnessing quantitative traits conferring partial resistance [29,30]. However, quantitative traits are highly dependent on environmental and experimental conditions, and the high variability in disease phenotyping among rice cultivars has hindered efforts to incorporate resistant traits using conventional breeding methods [29]. Breeding for disease resistance (or maintenance breeding) is often overlooked and undervalued by policymakers and producers as it does not raise the yield ceiling—only its floor. Producers tend to focus on cultivar yield potential (ceilings) instead of variability (floors) and, thus, may often undervalue the genetic resistance to a disease such as BPB that does not raise yield potential but raises the yield floor. In other words, although BPB resistance ultimately leads to an overall increase in net yield, it has not been of primary importance in breeding efforts due to emphasis on increasing the yield potential of new varieties [31–33]. Accordingly, this study attempts to estimate the value of raising the often overlooked “yield floor” through incorporation of potential BPB resistance in rice cultivars in the US Mid-South.

If commercial rice cultivars become BPB resistant, producers will benefit from their higher yields and global consumers will benefit from lower market rice prices. Simultaneously, there would be a likely increase in environmental efficiencies due to lower input requirements per unit of output (kg/rice). In addition to these benefits, rice yield potential is slowing, which demonstrates the need to focus on the floor rather than the ceiling in order to meet rising demand for rice. A meta-analysis [34] analyzed 13 global case studies in rice-growing environments to estimate and compare growth in yield potential attributed to rice breeding. They found the average growth rate associated with genetic gains to be 0.8% annually. While there is empirical evidence for rice yield gains overall, [34] concluded that recent progress in genetic gains is “definitely lacking.” Accordingly, breeding for resistance to biotic stresses like BPB is one way to increase the global rice supply without increasing genetic yield potential, since yield potential has become increasingly difficult to consistently obtain.

While the literature is rich on potential yield losses from BPB epidemics, there is a lack of research on the in-field economic and environmental damage caused by actual BPB occurrences. This study, thus, asks the counterfactual question: what benefits would be realized by producers, consumers, and the environment if all rice cultivars in the US Mid-South region were BPB resistant? To answer this question, this study (i) collected data on county- or parish-level rice cultivar yields (with associated BPB susceptibility ratings) and seeded areas in Arkansas, Louisiana, and Mississippi for 2003–2013, (ii) simulated BPB occurrence rates based on thresholds for disease onset found in the literature related to temperature and relative humidity using panicle emergence dates and county-level daily weather data for the entire region, (iii) simulated affected hectares as well as simulated yield loss based on historical BPB yield-loss data, and (iv) repeated steps ii and iii under +1°C, +2°C and +3°C warming scenarios. Based on the occurrence, yield loss, and warming scenario analysis, the study then estimated the additional rice volume that would have been in the market in the absence of BPB for 2003–2013, and further projected the implications of BPB for the global rice market. The study used a partial, spatial equilibrium model of the global rice economy to assess the market impact (e.g., prices, area, production and consumption, and producer and consumer welfare) of BPB in the US Mid-South. Lastly, the study analyzed how the counterfactual increased yield through BPB resistance would affect environmental impacts using several important metrics in a Life Cycle Assessment (LCA). Thus, the goals of this study were to estimate the producer, consumer and environmental impacts of BPB and how those impacts change under warming scenarios.

## Materials and methods

In continuation of previous studies [5,6,35], the current study estimates the potential benefits of BPB resistance for rice production in Arkansas, Louisiana, and Mississippi, which accounted for 68% of the total (long, medium, and short grain) 2017 US planted rice area [36,37]. The dataset consists of 34 rice cultivars, 33 rice-growing counties in Arkansas, 35 parishes in Louisiana, and 18 counties in Mississippi, for a total of 4,382 yield observations. Cultivar yields and BPB susceptibility ratings were obtained from university cultivar test plots across the region. The most reliable sources of relative yields are cultivar trials outside of actual farm observations [38,39]. The current study is based on previous data so some years had missing data on county- or parish-specific cultivar yields from experiment stations. In these cases, the annual county or parish average yield was used for that year. Data on actual annual cultivar planting areas were collected from the Rice Technical Working Group [40] for each rice-growing county and parish in different US Mid-South statesviz. Arkansas, Louisiana, and Mississippi from 2003 to 2013 (S1 Table). Arkansas and Mississippi typically plant in April or May and harvest between September and November. Louisiana typically plants between March and May and harvests between August and October (S2 Table shows the average day-of-year emergence and heading dates for the study) [40].

### Bacterial Panicle Blight resistance ratings for cultivars

BPB susceptibility ratings for each cultivar were obtained from historical observations of university-run experiment station (AAES, LSU AgCenter, MAFES) test plots in each state. The experiment stations used a Likert scale to rate the BPB susceptibility of rice cultivars as Moderately Resistant (MR), Moderately Susceptible (MS), Susceptible (S), and Very Susceptible (VS). S2 Table indicates from the previous test plot data that in the US Mid-South 2013 production year, the total area (hectares) of rice harvested from MR, MS, and S cultivars was 46%, 13%, and 41%, respectively [18,40]. Given that resistance to BPB can break down over time, the current study used the most recent ratings for each production year.

### Estimating Bacterial Panicle Blight occurrences

Although university extension services provide details on how to identify BPB in the field, they do not keep annual detailed accounts of county-level occurrences of the disease. To our knowledge, there are no extensive databases of locations, areas affected, nor total area of BPB occurrences in the US Mid-South. Existing literature suggests that BPB occurrences are triggered by conditions of high daily minimum temperatures in combination with simultaneously high relative humidity during the flowering stage. Specifically, this study uses 22 °C minimum daily temperature and 80% relative humidity based on previous findings [11,20,21,26]. BPB was commonly found when relative humidity was over 95% for 24 hours during flowering [11]. A study in Japan found that BPB developed when minimum daily temperature was ≥ 23° C and moderate rainfall (< 30 mm/day) occurred during the rice-heading stage. In their comprehensive in-field study, [20] found that BPB was not present when the minimum daily temperature was below 22°C and daily mean relative humidity was lower than 80% for the period of seven days from the first panicle emergence through the heading stage. The range of relative humidity needed for BPB to develop is more nebulous, [15] found that BPB occurred when relative humidity was between 75% and 95%.

PRISM weather data was collected by county for 2003–2013, derived from a 4 km grid, and includes maximum and minimum temperatures as well as vapor pressure deficit [41]. Relative humidity was calculated using the “plantecophys” package in R Statistical Software [42]. Using historical daily weather data from 2003 to 2013 for the seven days after panicle emergence, the current study estimated the location and year of BPB occurrences at the county/parish level when minimum daily temperatures and daily relative humidity thresholds were met (S1 and S2 Figs). Thus, at the county/parish level, the current study predicts an occurrence as follows:

$$g(x)=\{\begin{array}{c} 1\;if\;{RH}_{lt}>TRH⋂{AMTemp}_{lt}>TTemp\\ 0\;Otherwise\; \end{array},$$ (1)

$${Occurrence}_{lt}=g(x)=1$$

where *RH*_*lt*_ is the average daily relative humidity in county/parish *l* seven days surrounding panicle emergence in year *t*. *AMTemp*_*lt*_ corresponds to the average minimum daily temperature seven days surrounding panicle emergence in year *t* in county/parish *l*. *TRH* is the threshold of relative humidity which can vary between 75% and 80% based on the minimum RH percentage required for a BPB occurrence, respectively [15,20]. In this study, occurrences are modeled where *TRH* is equal to or greater than 77%. *TTemp* is the threshold temperature, which is 22°C, as identified by [20]. Thus, if both thresholds (temperature and relative humidity) are met, the model predicts *g(x)* to be “1,” which indicates a BPB occurrence in county *l* in year *t* that would affect all rice cultivars in county *l*.

It is unrealistic to assume that the climatic thresholds in Eq (1) will lead to a 100% BPB infection in rice fields and cause subsequent yield loss. Specifically, the levels of pathogen inoculum and the timing of infection are relevant for yield loss, which must be accounted for in estimating BPB occurrences. In their study, [21] found that on average 31% of diseased panicles actually resulted in yield loss. As such, a large number of fields with the same susceptible cultivars would not become infested with yield loss even having reached the climatic threshold. In this study, it is instead assumed that 31% of the area in which the climatic threshold is reached in Eq (1) are associated with a yield loss. Still, this is a simplistic assumption because the study assumes all rice fields planted to a particular cultivar with unique susceptibility, tolerance, and resistance levels experience the same percentage yield loss from BPB for the set of climatic conditions. However, to model the potential effects of BPB on a macro level, assumptions like those made in this study are a necessity until more detailed data collection becomes possible.

### Bacterial Panicle Blight field trials for yield loss

If an occurrence is predicted in Eq (1) for a given county/parish for a given year, yield losses will vary by each cultivar’s yield potential and its respective BPB susceptibility rating. To illustrate, if two cultivars have the same BPB susceptibility ratings but different yield potentials (or vice versa) then yield loss should be different between the two cultivars. The yield loss percentage for each susceptibility rating (MR, MS, S, and VS) was derived from data collected from six field trials conducted in Eagle Lake and Beaumont, Texas in 2010 and 2011 [17]. In each of the trials, rice cultivars and elite breeding lines were arranged in a randomized complete block design with three or four replications. Plots consisted of six 2.7-m rows, spaced 0.19 m between rows, for the Eagle Lake trials and seven 2.7-m rows, spaced 0.18 m between rows, for the Beaumont trials. Rice was drill seeded, and all agronomical, weed and insect management followed local practices. Plots were spray inoculated with the *B*. *glume* pathogen before the flowering stage. BPB severity was rated by the maturity of each cultivar or breeding lines on a scale of 0 to 9, where 0 represents no symptoms and 9 represents most severe in symptoms and damage to panicles. Plots were harvested at the maturity of each cultivar or breeding line using a plot combine and grain yield adjusted to 12% grain moisture.

### Market impacts of Bacterial Panicle Blight

The total cost of a BPB occurrence in year (*t*), *TC*_*t*_, is modeled in Eq (2):

$${TC}_{t}=\sum_{l,i}(\gamma_{lt}{0.31A}_{ilt}[\delta_{i}Y_{ilt}P_{it}]),$$ (2)

where *γ*_*lt*_ is a dummy variable for the exceedance threshold in Eq (1) for county/parish (*l*) in year (*t*). *γ*_*lt*_ = 0 when threshold is not exceeded and no BPB occurrence, and *γ*_*lt*_ = 1 when threshold is exceeded and disease onset occurs. The coefficient 0.31 represents the share of susceptible rice hectares that become infested when a BPB onset occurs, *A*_*ilt*_ is the sum of all historic hectares of BPB susceptible rice cultivars (*i*) sown in each rice producing county/parish (*l*) in year (*t*), *δ*_*i*_ is the yield loss associated with BPB ratings (MR, MS, S, and VS) by cultivar (*i*), *Y*_*ilt*_ is the yield by cultivar (*i*), county/parish (*l*), and year (*t*), and *P*_*it*_ is the season-average farm price by rice cultivar (*i*) and year (*t*). *P*_*it*_ was measured in $/metric ton ($/mt) and aggregated at the grain-type level (long grain and medium grain) as reported by [37]. The current study used the average yield loss for long grain and medium grain rice due to BPB for the period 2003–2013 to investigate the market impacts of BPB. Specifically, the study used a spatial, partial, supply-chain model of the global rice economy [43] to assess the impact of the following counterfactual scenario: “what would have been the impact of a BPB occurrence in the US Mid-South in 2013–2013 on the US and global rice market?” The model calibrates to the market conditions of the 2013–2015 period, and the global rice economy is disaggregated into 76 regional markets and nine rice commodities derived from a combination of rice type (long, medium, and fragrant rice) and milling degree (paddy, brown, and milled rice). This high level of disaggregation allows for the analysis of the impact of BPB on the prices consumers pay in local markets. See S1 Appendix for details on the modeling framework.

### Environmental impacts of Bacterial Panicle Blight

An LCA was conducted to quantitatively compare the cradle-to-farm gate environmental impact of BPB following an approach similar to previous work [5,35,44]. An evaluation was conducted for counterfactual scenarios that consider the elimination of BPB under current climatic conditions and under a warming scenario with a 1°C increase in daily temperature. The functional unit is 54 kg per year, the global per capita rice consumption, which serves as the basis for comparative evaluation. Each scenario includes attributional CO_2_ emissions arising from indirect land use change [45].

The rice yield (kg/ha) associated with no BPB, with current BPB, and with BPB under 1°C warming, respectively, are used to simulate and compare environmental impact scenarios. Pesticide and herbicide usage are assumed to be the same across all scenarios. Fuel and water use are considered equivalent across production systems. Inputs for each scenario were adapted from the University of Arkansas Extension budgets [46]. Similar to Durand-Morat et al. 2018, this study used the Stepwise Life Cycle Impact Assessment framework, which combines human and environmental effects in an economic valuation scheme [35,47,48]. The cost of environmental externalities is accounted for with a consistent structure [49]. The lifecycle impact categories included in the stepwise method are described in S4 Table. Midpoint and endpoint characterization factors are provided [48,50]. Normalization and weighting factors based on 1995 European Union per-capita emissions are given. The Stepwise method basis damage characterization in a fashion to account for both human health and the ecosystem quality. Effects to human health are quantified by quality-adjusted life years (QALY), a measure of costs associated with morbidity and mortality, and ecosystem quality is quantified by biodiversity-adjusted hectare years (BAHY), a measure of costs associated with biodiversity loss. Costs associated with QALY and BAHY are calculated based on contributing factors to the midpoint impact categories. Using a budget constraint argument and an estimate of average global income, it is argued that the maximum average funds available to reach full-quality of human life in a year is 72,776 (2017 USD) [48]; further, one BAHY is equivalent to 1/14 QALY [48,51]. The results presented as costs can be interpreted as the estimated expense to balance the environmental and human health externalities, that is, to restore full QALYs and BAHYs based on the “ability to pay” [49].

## Results

### Yield loss in field trials

Table 1 shows each cultivar in the field trials and its assigned BPB susceptibility rating. Table 1 also shows each cultivar’s “yield potential”, which was derived from the cultivar yield trials (i.e. straight yield trials) for the same cultivars at the same station for each year. The percent difference between the yield potential and the yield when inoculated with BPB represents the yield reduction, “% Yield Loss,” associated with BPB. Table 1 indicates that on average MR, MS, S, and VS cultivars lose 17.7%, 21.0%, 25.0%, and 37.4% of their yield potential, respectively, when infected with BPB. This range is similar to that of [15,16], who found a 40% yield loss in Louisiana for severely infected fields planted to highly susceptible varieties. Thus, if both thresholds are met in Eq (1) for county/parish *l* in year *t*, then yield penalty (δ) would be applied to each cultivar based on its BPB susceptibility rating (MR: 17.7%, MS: 21.0%, S: 25.0%, and VS: 37.4%) and each cultivar’s county/parish level yield potential.

**Table 1:** Susceptibility to Bacterial Panicle Blight, average rice yields, and estimated yield loss due to Bacterial Panicle Blight by variety in field trials.

Cultivar	Average BPB severity (0 to 9)^a^	BPB susceptibility rating^b^	Average yield (kg/ha)	Yield potential (kg/ha)^c^	% Yield loss
Catahoula	2.3	MR	9,188	10,050	8.58%
CL 151	2.7	MR	8,960	10,260	12.67%
Presidio	2.3	MR	5,938	7,151	16.96%
RU0803092	2.7	MR	8,316	10,766	22.76%
RU0803190	2.7	MR	6,778	8,141	16.74%
Taggart	2.0	MR	7,045	9,872	28.64%
Bowman	3.3	MS	6,043	6,852	11.81%
Cheniere	4.0	MS	7,402	8,424	12.13%
CL 111	3.7	MS	8,068	9,198	12.28%
CL 181- AR	4.3	MS	4,631	6,340	26.94%
Francis	4.3	MS	6,686	8,289	19.34%
Jupiter	3.3	MS	6,911	8,320	16.93%
Neptune	3.0	MS	6,425	8,422	23.72%
Rondo	3.7	MS	7,770	9,365	17.03%
RU0703184	3.3	MS	6,631	8,950	25.91%
RU0803116	3.7	MS	6,334	8,581	26.19%
RU0803181	4.3	MS	6,762	8,020	15.68%
RU0903184	4.3	MS	6,381	10,245	37.71%
Sabine	4.3	MS	6,649	10,469	36.49%
Wells	3.7	MS	8,400	9,525	11.81%
Cocodrie	5.3	S	7,169	8,114	11.65%
RU0703147	6.0	S	7,845	9,665	18.83%
Templeton	5.7	S	5,224	9,402	44.43%
CL 142-AR	8.3	VS	4,413	7,488	41.07%
CL 261	8.7	VS	4,745	6,313	24.84%
Jazzman	7.7	VS	3,773	7,015	46.22%
Susceptibility Rating^b^	Average % Loss				
VS: Very Susceptible	37.4%				
S: Susceptible	25.0%				
MS: Moderately Susceptible	21.0%				
MR: Moderately Resistant	17.7%				

^a^BPB severity was rated near maturity on a scale of 0 to 9 where 0 represents no symptoms, and 9 represents most severe in symptoms and damage to panicles.

^b^ Susceptibility ratings are subjectively idenitified as VS, S, MS, and MR based on the Likert scale, and in these field trials the average percentage yield loss is quantified by subjective susceptibility rating in order to estimate BPB yield losses by susceptibility rating [17].

^c^ Average yields represent yields without any BPB infestation.

### Producer impacts

In S3 Fig, the average annual rice hectares planted are presented alongside the average county-level hectares planted to susceptible varieties (MS, S, VS). The spatial distribution of hectares planted to susceptible varieties is relatively homogenous across years at the county level, which likely indicates that major losses due to BPB are closely related to overall production. Table 2 presents the annual aggregate losses, in 2017 USD, from BPB occurrences. S5 Table indicates which counties/parishes and which years were estimated to have a BPB occurrence. A total of 162 county/parish occurrences from 2003–2013 were estimated using the threshold of the average minimum temperature seven days following panicle initiation being above 22°C and the average relative humidity for the same time period being above 77%. The more detailed state-level losses for medium and long grain are presented in S6 and S7 Tables. Similar to previous BPB scouting assessments [50], 2010 was the most severe occurrences both in terms of the number of incidents and total area affected. The model estimates that 2010 accounted for 28% of all county/parish occurrences estimated for the 2003–2013 period.

**Table 2:** Estimated total losses from bacterial panicle occurrences in the Lower Mississippi Delta Region: 2003–2013.

Year	Medium Grain Rice			Long Grain Rice			Total Production Loss
	Price	Production Loss		Price	Production Loss		
	$/MT^a^^,^^b^	1,000 MT^c^	$ million	$/MT^a^^,^^b^	1,000 MT^c^	$ million	$ million
2003	289.83	9.03	2.62	221.6	174.07	38.57	41.19
2004	207.02	6.44	1.33	208.45	112.97	23.55	24.88
2005	260.66	34.93	9.11	200.50	253.37	50.80	59.91
2006	321.95	0.33	0.11	251.97	24.17	6.09	6.20
2007	377.71	3.43	1.29	320.79	29.33	9.41	10.70
2008	453.43	0.00	0.00	412.32	0.00	0.00	0.00
2009	392.54	0.00	0.00	333.11	0.00	0.00	0.00
2010	368.98	60.12	22.18	287.77	277.64	79.90	102.08
2011	340.99	8.30	2.83	295.69	46.55	13.76	16.59
2012	343.43	21.91	7.53	320.60	164.01	52.58	60.11
2013	356.51	0.00	0.00	346.80	37.59	13.03	13.03
Total		144.49	47.00		1,119.71	287.70	334.70

^a^Prices and values expressed in 2017 USD.

^b^USDA reports medium grain prices from 2003–2008 as USA average and reports 2009–2013 as US Mid-South (Arkansas, Louisiana, Mississippi, Missouri and Texas) averages. Price data retrieved from [41].

^c^Summation of medium and long grain losses from Arkansas, Louisiana and Texas found on S6 and S7 Tables, respectively.

The average annual loss attributed to BPB occurrences in the US Mid-South was estimated at 30.42 million USD between 2003 and 2013. In some years (2008 and 2009), there were no estimated losses, and in others (2005, 2010, and 2012), losses were estimated to be over 59 million USD. It is worth noting that the economic damage of an occurrence is a function of not only the presence of the disease itself but also the area it affects and the price of rice for a given year. Moreover, the total damage estimated to the US Mid-South rice producers from BPB from 2003 to 2013 was estimated to be 334.70 million USD. The results from S6 and S7 Tables indicate that on average between 2003 and 2013, the loss from BPB accounts for 2.13% and 1.61% of the total medium and long grain rice production, respectively.

### Predicted yield losses based on a warming scenario

To evaluate the effect of increasing temperatures, 1°C was uniformly added to the observed daily weather dataset from 2003 to 2013 to estimate the change in predicted occurrences given the same thresholds (minimum average temperature seven days surrounding panicle emergence at ≥ 22°C, and average daily relative humidity seven days surrounding panicle emergence at ≥ 77%). At the county/parish level, Louisiana experiences more extensive damage under the warming scenario but in the same parishes as in the 2003–2013 period, while Arkansas and Mississippi experience increased damage in the same counties and more widespread occurrences in other counties.

A total of 315 county/parish BPB occurrences from 2003 to 2013 were estimated under the 1°C warming scenario, which is an increase of 94% from the actual observed temperatures. Table 3 indicates that total losses under the 1°C warming scenario increase to 503.94 million USD from 2003 to 2013, averaging 45.81 million USD in economic losses per year. The 1°C warming scenario suggests that the increase in BPB and its subsequent yield losses would have resulted in 51% higher total production losses than those estimated in the baseline. Notably, all years were affected by the warming scenario, and some warmer years (2004, 2005, and 2007) saw larger increases of estimated BPB incidences due to the temperature increase. Although the increase in temperature was linear, the estimated damages are nonlinear due to heterogeneity in county/parish rice acreage and spatial temperature distribution around the threshold. The disproportionate increase in total economic damages reported in Arkansas under the warming scenario is explained by the fact that Arkansas produces more than 40% of the total US rice crop and, as such, has more area that could experience yield losses. Regardless of the spatial aspects of the losses, it appears that a marginal increase in temperature could have relatively large impacts on the economic damage associated with the disease.

**Table 3:** Estimated total losses from bacterial panicle occurrences given an uniform 1°C increase in temperature in the Lower Mississippi Delta Region: 2003–2013.

Year	Medium Grain Rice			Long Grain Rice			Total Production Loss
	Price	Production Loss		Price	Production Loss		
	$/MT^a^^,^^b^	1,000 MT^c^	$ million	$/MT^a^^,^^b^	1,000 MT^c^	$ million	$ million
2003	289.83	12.53	3.63	221.6	238.68	52.89	56.52
2004	207.02	10.02	2.07	208.45	214.36	44.68	46.76
2005	260.66	37.05	9.66	200.5	383.71	76.93	86.59
2006	321.95	1.93	0.62	251.97	61.37	15.46	16.08
2007	377.71	24.42	9.23	320.79	161.00	51.65	60.87
2008	453.43	4.35	1.97	412.32	29.83	12.30	14.27
2009	392.54	11.23	4.41	333.11	25.68	8.56	12.97
2010	368.98	50.92	18.79	287.77	292.77	84.25	103.04
2011	340.99	8.84	3.02	295.69	49.02	14.49	17.51
2012	343.43	22.05	7.57	320.6	186.48	59.78	67.36
2013	356.51	1.31	0.47	346.8	62.03	21.51	21.98
Total		**184.64**	**61.43**		**1,704.92**	**442.51**	**503.94**

^a^Prices and values expressed in 2017 USD.

^b^USDA reports medium grain prices from 2003–2008 as USA averages and reports 2009–2013 as US Mid-South (Arkansas, Louisiana, Mississippi, Missouri and Texas) averages. Price data retrieved from [41].

^c^Summation of losses from Arkansas, Louisiana and Texas found on S6 and S7 Tables.

**Fig 1:**
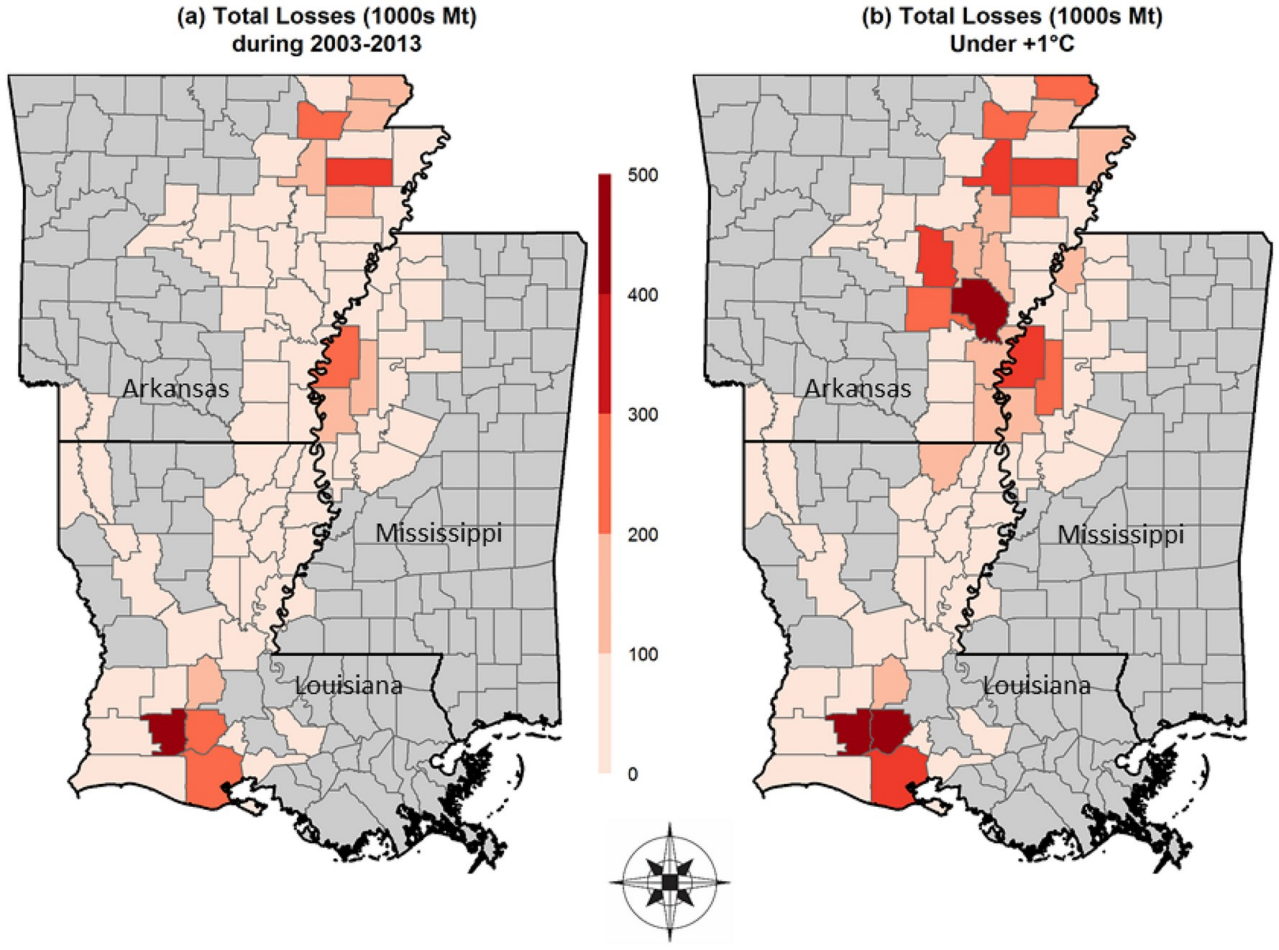
County-level rice production losses to Bacterial Panicle Blight (BPB) in Arkansas, Louisiana, and Mississippi.

Following the +1°C methodology described above we analyze the effects of +2 and +3°C warming scenarios. Fig 1 shows the economic impacts, via yield losses, and highlights the non-linearity in the impact of +1°C warming. The non-linearity in losses can be attributed to the distribution of temperatures and resulting “clustering” of temperatures around certain thresholds but not others. The average yearly losses associated with a +2°C and +3°C were estimated to be 68.94 and 85.35 million USD, respectively (Table 4). These results would seem to indicate that BPB will become more prevalent and damaging in a warming climate.

**Table 4:** Estimated economic impact of a Bacterial Panicle Blight (BPB) occurrence under current (BPB) and 1°C warming conditions (BPB 1°C) on selected U.S. market variables relative to the baseline market average in 2013–2015.

	Total: Long and Medium Grain Rice				
	Baseline	Change by scenario (%)			
		BPB	BPB 1°C	BPB 2°C	BPB 3°C
Production paddy rice (tmt)	6,509	-1.2%	-1.8%	-2.6%	-3.3%
Demand milled rice (tmt)	3,974	0.0%	0.0%	0.0%	-0.1%
Exports (tmt)	3,164	-2.3%	-3.4%	-4.9%	-6.2%
Imports (tmt)	715	0.8%	1.3%	1.8%	2.2%
Producer paddy price ($/mt)	332	1.4%	2.1%	3.1%	3.9%
Retail price ($/mt)	1,813	1.0%	1.5%	2.2%	2.9%
Value production ($ million)	3,084	0.2%	0.3%	0.5%	0.6%
Value Consumption ($ million)	7,209	1.0%	1.5%	2.2%	2.8%
Producer surplus ($ million)	-	4.8	7.2	10.4	13.1
Consumer surplus ($ million)	-	-69.0	-112.0	-160.0	-204.0
Rice area (1,000 ha)	1,117	0.08%	0.10%	0.14%	0.16%

### Market impacts of Bacterial Panicle Blight

The current study simulated the economic impact of BPB by shocking rice yields based on the losses estimated in the section above, under the assumption that BPB did not affect the yields obtained during the benchmark period 2013–2015. Table 4 demonstrates the market impacts of BPB on rice production in the US Mid-South. An average BPB occurrence in the US generates a new market equilibrium that results in production losses of 1.2% (79 tmt/year), primarily of long grain rice (1.5% or 72 tmt/year) and, to a lesser extent, medium grain rice (0.4% or 7 tmt/year), and increases in rice area by 0.06% (651 ha). The decrease in productivity due to BPB increases production costs and prices along the US rice supply chain, undermines the competitiveness of US rice, and decreases US rice exports by 2.3% (72 tmt/year), which breaks down into 2.8% (65 tmt) for long grain rice and 0.8% (7 tmt) for medium grain rice. Due to the inelastic nature of rice demand in the US, the increase in rice producer prices (1.8% for long grain and 0.4% for medium grain rice) more than offsets the decrease in rice production, which increases producer welfare slightly by $4.8 million (2018 USD). On the other hand, consumer welfare decreases by $69.0 million as rice market prices increase due to BPB occurrences.

A 1°C warming scenario generates a new market equilibrium with production losses in the US Mid-South estimated to reach 1.8% or 117 tmt/year (2.2% or 108 tmt/year for long grain and 0.5% or 9 tmt/year for medium grain rice), and require 0.10% or 1,000 ha more than in the benchmark scenario. Exports of US rice decrease by 3.4% or 107 tmt/year (4.3% or 98 tmt/year for long grain and 1.0% or 9 tmt/year for medium grain rice). Producer prices for long grain and medium grain rice increase by 2.7% and 0.5%, respectively, which more than offsets the decrease in rice production and results in a $7.2 million increase in producer welfare. Consumer welfare is estimated to decrease by $112.0 million as rice market prices increase due to BPB under a 1°C warming scenario. The 2°C and 3°C warming scenarios further reduce rice production efficiency, which results in lower rice supplies and exports, larger rice area, higher producer and consumer prices, and higher (lower) producer (consumer) welfare.

These results suggest that the reduction in US rice exports attributed to BPB is sufficient to feed 1.46 million people every year at the average global rice consumption rate of 54 kg per person annually, and 2.17, 3.11, and 3.98 million people under the 1°C, 2°C, and 3°C warming scenarios, respectively. This finding is substantial considering the US is a relatively small rice producer by global standards.

### Environmental impacts of Bacterial Panicle Blight

The current study evaluated the environmental impacts associated with the BPB infection in rice through a counterfactual argument. Specifically, the current production (with BPB) was compared to the current counterfactual (absence of BPB induced yield loss). The difference between the two scenarios is manifested as a yield gain for the counterfactual case of 2.47% for current conditions and 4.58% under future conditions assuming a 1°C increase in average temperature. Table 5 and S8 Table present the numerical results for each of the Stepwise impact category. The single score is the sum of estimated external costs associated with impacts that are associated with the full production supply chain for the average annual global consumption, 54 kg. In Table 5, respiratory inorganics and effects associated with global warming (increased rates of disease and impacts on agricultural production) are the major contributors to external environmental costs associated of rice production. For the U.S. conditions evaluated, there would be an avoided external cost of $0.42 and $0.78 per person per year for the two BPB free scenarios, respectively. This translates to potentially avoided costs of $1.28 and $5.32 million annually for the current counterfactual and climate change scenarios, respectively. These are the external costs in addition to loss of economic revenue resulting from yield reduction induced from BPB.

**Table 5:** Environmental impact scores using Stepwise LCA method.

Impact category	Unit	Baseline	Panicle Resistant	Panicle Resistant plus 1°C
**Endpoint Impact Scores**				
Single Score	US$ 2019	$ 27.45 (6.9%)	$ 27.03 (6.9%)	$ 26.67 (6.9%)
Global warming, fossil	US$ 2019	$ 14.14 (8.6%)	$ 14.00 (8.6%)	$ 13.88 (8.7%)
Respiratory inorganics	US$ 2019	$ 9.70 (6.8%)	$ 9.50 (6.8%)	$ 9.32 (6.8%)
**Midpoint Impact Scores**^a^				
Global warming, fossil	kg CO2-eq	113 (8.6%)	112 (8.6%)	111 (8.7%)
Respiratory inorganics	kg PM2.5-eq	0.1 (6.8%)	0.09 (6.8%)	0.09 (6.8%)
Photochemical ozone, vegetation	m2*ppm*hr	2069 (8.5%)	2038 (8.5%)	2010 (8.5%)
Eutrophication, terrestrial	m2 UES	30.11 (5.9%)	29.66 (5.9%)	29.27 (5.9%)
Human toxicity, non-carc.	kg C2H3Cl-eq	0.74 (92%)	0.66 (101%)	0.6 (111%)
Ecotoxicity, aquatic	kg TEG-eq w	40815 (15.%)	40705 (15.1%)	40609 (15.1%)
Ecotoxicity, terrestrial	kg TEG-eq s	192 (28.4%)	189 (28.7%)	187 (28.9%)
Human toxicity, carcinogens	kg C2H3Cl-eq	0.61 (9.3%)	0.6 (9.3%)	0.59 (9.3%)
Nature occupation	m2-years ag	1.17 (15.2%)	1.15 (15.2%)	1.13 (15.1%)
Eutrophication, aquatic	kg NO3-eq	0.63 (13.6%)	0.62 (13.6%)	0.62 (13.6%)
Acidification	m2 UES	7.57 (6.8%)	7.46 (6.8%)	7.36 (6.8%)
Global warming, non-fossil	kg CO2-eq	5.92 (19.%)	5.92 (18.9%)	5.93 (18.8%)
Respiratory organics	pers*ppm*hr	0.21 (9.4%)	0.2 (9.5%)	0.2 (9.5%)
Mineral extraction	MJ extra	2.88 (11.4%)	2.81 (11.5%)	2.75 (11.5%)
Ozone layer depletion	kg CFC-11-eq	1.8E-6 (31.1%)	1.8E-6 (31.1%)	1.8E-6 (31.1%)
Non-renewable energy	MJ primary	858 (13.8%)	841 (13.8%)	826 (13.9%)

The single score is the sum of monetary cost of all impact categories. Only the two most costly are shown individually. Values in parentheses are coefficients of variation based on 1000 Monte Carlo Simulation runs.

^a^Economic cost for each category below respiratory inorganics is less than USD2019 1.20.

S8 Table presents an alternative view of the impact scores based on normalizing the contribution from rice consumption against the cumulative total impact for each category. Thus, from this perspective, it can be inferred that production of rice contributes approximately 11 percent of combined eco-toxicity potential and less than about 3 percent of per capita annual impact of the remaining categories. To quantify the uncertainty of these results, the Simapro modeling platform was used to perform Monte Carlo simulations (MCS) using the available uncertainty information to propagate the input uncertainty to output uncertainty. These results are presented as coefficients of variation for each impact category in Table 5.

Table 5 suggests overlapping distributions, the differences in mean values were constructed in a pairwise manner where each random variate in baseline yield of the Monte Carlo simulation was parametrically linked to the yield in the alternate scenario ensuring that the alternate had a larger yield (Eq 3):

$$Y_{a}=\frac{Y_{b}}{\left(1-{VAR}_{Y}\right)},$$ (3)

where *Y*_*a*_ is alternate yield, *Y*_*b*_ is the MCS variate for baseline yield, and *VAR*_*Y*_ is the random variate for yield loss of the baseline compared to the alternate drawn from the probability density function shown in S4 Fig. The overlap is also demonstrated in S5 Fig, which presents the difference in a calculated single score (2018 USD) for the current baseline and current counterfactual scenario for 10,000 MCS runs to provide a smooth distribution.

The results from this study indicate that the alleviation of the BPB disease from even a relatively small global rice producer like the US can have far-reaching implications for rice producers and consumers. It is important to better understand the thresholds that trigger the disease and subsequent economic damage. For a comparison of these results to other prevalent rice diseases, [6] found that sheath blight caused 890.25 million dollars (68.48 million annually) of damage for the same location and time period. [5] found that rice blast caused 901.45 million dollars (69.34 million annually) of damage for the same location and time period. Unlike the [5,6] studies, which found that rice blast and sheath blight, respectively, had large and relatively consistent damage across time, this study shows that BPB seems to be more prone to sporadic but more substantial losses. Unlike rice blast and sheath blight occurrences that can be partially mitigated with fungicide applications, once the onset of BPB occurs there is no effective chemical application option available that can dampen its effects.

## Discussion

In this study,macro-level effects of BPB were estimated for Mid-South US rice production for 2003–2013 and for simulated future warming. Moreover, the modeling technique implemented in this study is unique as it pieces together parameters from field experiments with county/parish-level data on BPB susceptibility ratings for area planted by cultivar. Given the potential threat of BPB and its effect on global food security, this study makes a first attempt at moving from local field studies to estimating the global implications of BPB. Scaling field experiments up to country-level analysis is fraught with issues that this study attempted to mitigate. Selecting a single threshold as a condition for disease occurrence, as was conducted in this study, is an oversimplification of any biological process. Variations in pathogen and host population and agricultural practices bring the need for further in-field evaluations. Also, it should be recognized that rainfall is not the only reason for elevated levels of relative humidity, and the relative humidity is not the reason for elevated levels of disease. Under specific conditions surface wetness is formed which provides necessary conditions for the infection [52].

Future field research might consider the timing of the weather thresholds within the growing season. Two consecutive days of high temperature and high relative humidity exposure might increase the probability of occurrence of BPB more than a simple average of the seven days surrounding panicle emergence. Further research is also needed to better understand the magnitude of threshold values under which the disease is present in field conditions. Specifically, the relationship between temperature, relative humidity, and BPB is likely to be more complex than modeled here and is probably impacted by the rice type (hybrid vs inbred), planting date, growth stage, soil type, and the interaction of other weather variables including canopy temperature, canopy relative humidity, wind speed, and solar radiation. Additionally, research could devote more attention to improving specifications of the magnitude, duration, and frequency of extreme (hot and humid) weather events in BPB occurrences. In the near future, there may be opportunities to improve in-field monitoring and tracking of diseases like BPB, among others, especially where continuous field trials are conducted on rice around the world. In many regions, field trials exclude critical information on location (coordinates), metadata on crop phenology (e.g., time of panicle emergence), and potential areas and times of disease occurrences. Equipped with such information, occurrence models, forecasts, and impact assessments could be greatly improved.

Knowledge of potential climate change impacts on the environmental and economic variables, such as production efficiency, ecotoxicity, and shifts in international trade due to price and volume effects, provide a dual argument for renewed and increased support for BPB-resistance research in breeding programs. Moreover, future research could focus some attention on the effects of BPB on grain quality and nutritional attributes. Understanding BPB impacts on yield and quality are critical with regard to rice, given its importance as a staple crop in many low-income countries and projected growth in global populations in coming decades [53]. Additionally, as global warming intensifies, BPB could become a more regular and formidable rice disease to combat, and breeding for BPB resistance is likely the primary line-of-defense as no effective chemical options are currently available.

This study provides insights for policymakers, extension personnel, plant breeders, plant pathologists, rice producers, and rice consumers about how potential genetic resistance to BPB under current production and a future warming scenario could affect producer and consumer livelihoods, food security, and environmental sustainability (through reduced input use and subsequent ecotoxicity per kg of rice produced). Importantly, this study sets out to illustrate that as the rice yield gap closes and the yield ceiling approaches, maintenance breeding for disease resistance is one way to continue increasing the food supply despite low growth in yield potential.

## Conclusions

Climate change is expected to lead to increased temperatures in many regions of the world [54]. While humidity predictions are less certain, there will be regions with both increased temperature and humidity, which will lead to increased BPB infection pressure in rice production globally. Based on the results of this study, even marginal increases in temperature (+1°C) during the rice-growing season may cause significant economic impacts in the rice economy. Thus, with global warming, BPB could become one of the most economically destructive rice diseases within the next few decades. The current study showed that increased rates of BPB infection can have substantial economic, environmental, and food security costs, hence demonstrating the high value that should be placed on rice breeding efforts toward BPB resistance. Policymakers, agriculturalists, and economists may better forecast and plan for a resilient future in rice production and consumption with the findings of this study.

## Supporting information

S1 Fig Variability in minimum temperatures by State. (DOCX)

S2 Fig Variability in relative humidity by State. (DOCX)

S3 Fig Average annual hectares planted for rice production: 2003–2013. (DOCX)

S4 Fig Probability distribution for comparative Monte Carlo testing accounting for uncertainty in yield loss and inputs. (DOCX)

S5 Fig Probability density functions for yield loss simulation in Monte Carlo assessment of variability. (DOCX)

S1 Table Cultivar bacterial panicle blight rating, hectares, and yield by State. (DOCX)

S2 Table Average emergence and heading Day-of-Year by State, 2003–2013. (DOCX)

S3 Table Yearly rice area (hectares) sown to various levels of Bacterial Panicle Blight resistance. (DOCX)

S4 Table Environmental impact categories used in the Life Cycle Assessment (LCA) for a Bacterial Panicle Blight occurrence (Stepwise method). (DOCX)

S5 Table Estimated Bacterial Panicle Blight occurrence years and locations in the lower mississippi delta region: 2003–2013. (DOCX)

S6 Table Estimated state-level losses from BPB in medium grain rice production: 2003–2013. (DOCX)

S7 Table Estimated state-level losses from BPB in long grain rice production: 2003–2013. (DOCX)

S8 Table Normalized environmental impact scores. The Values Represent the Percent of Cumulative Annual (EU) Impact in Each Category Attributable to Rice Consumption.
(DOCX)

S1 Appendix Description of the partial spatial equilibrium model of the global rice economy. (DOCX)
