# Supplementary figures and images for: Warming increases Bacterial Panicle Blight (Burkholderia glumae) occurrences and impacts on USA rice production
Source: PLoS One. 2019 Jul 11;14(7):e0219199. doi: 10.1371/journal.pone.0219199 (PMC6623956; doi:10.1371/journal.pone.0219199)

**
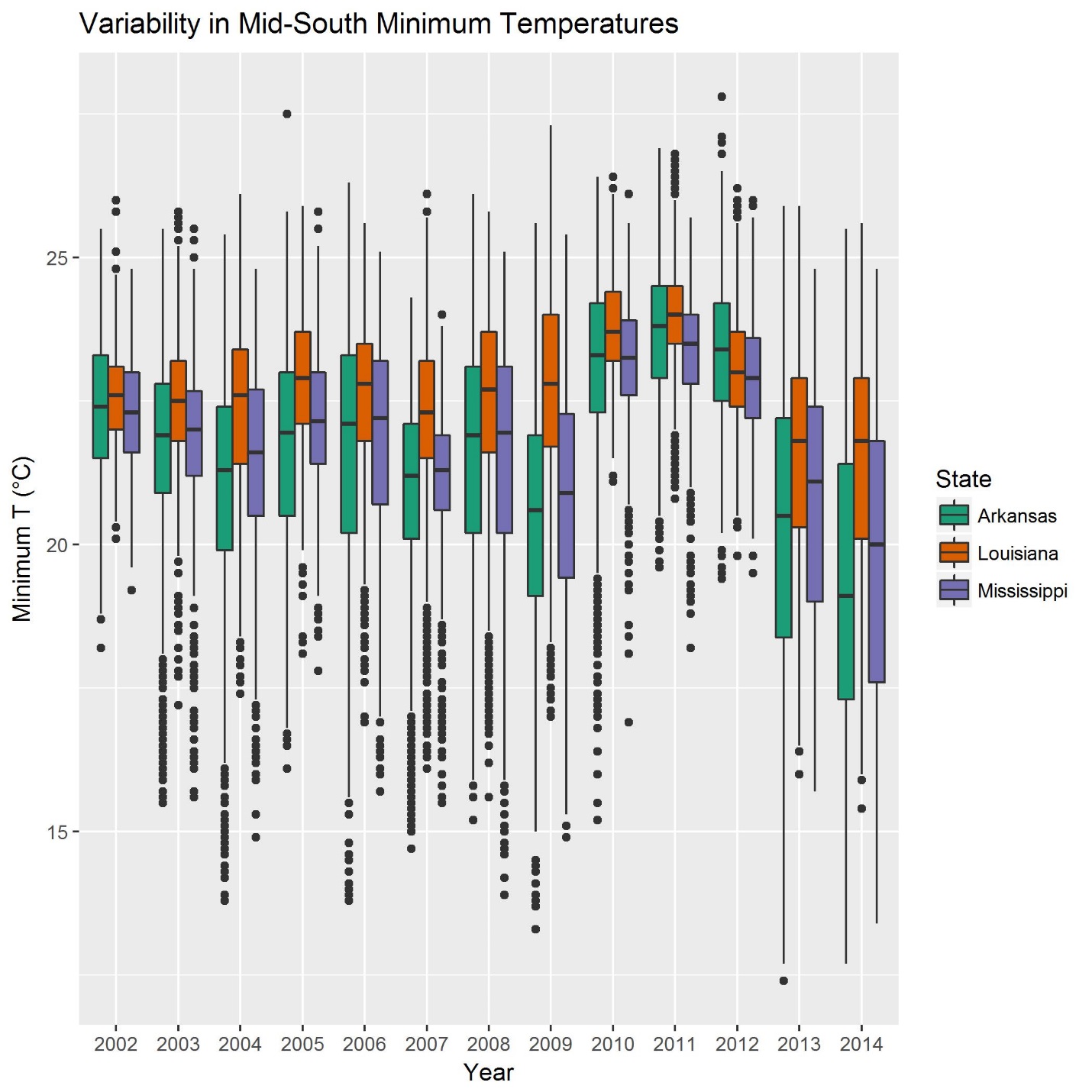
**

Supplement: S1 Fig — (DOCX) [file pone.0219199.s001.docx]

**
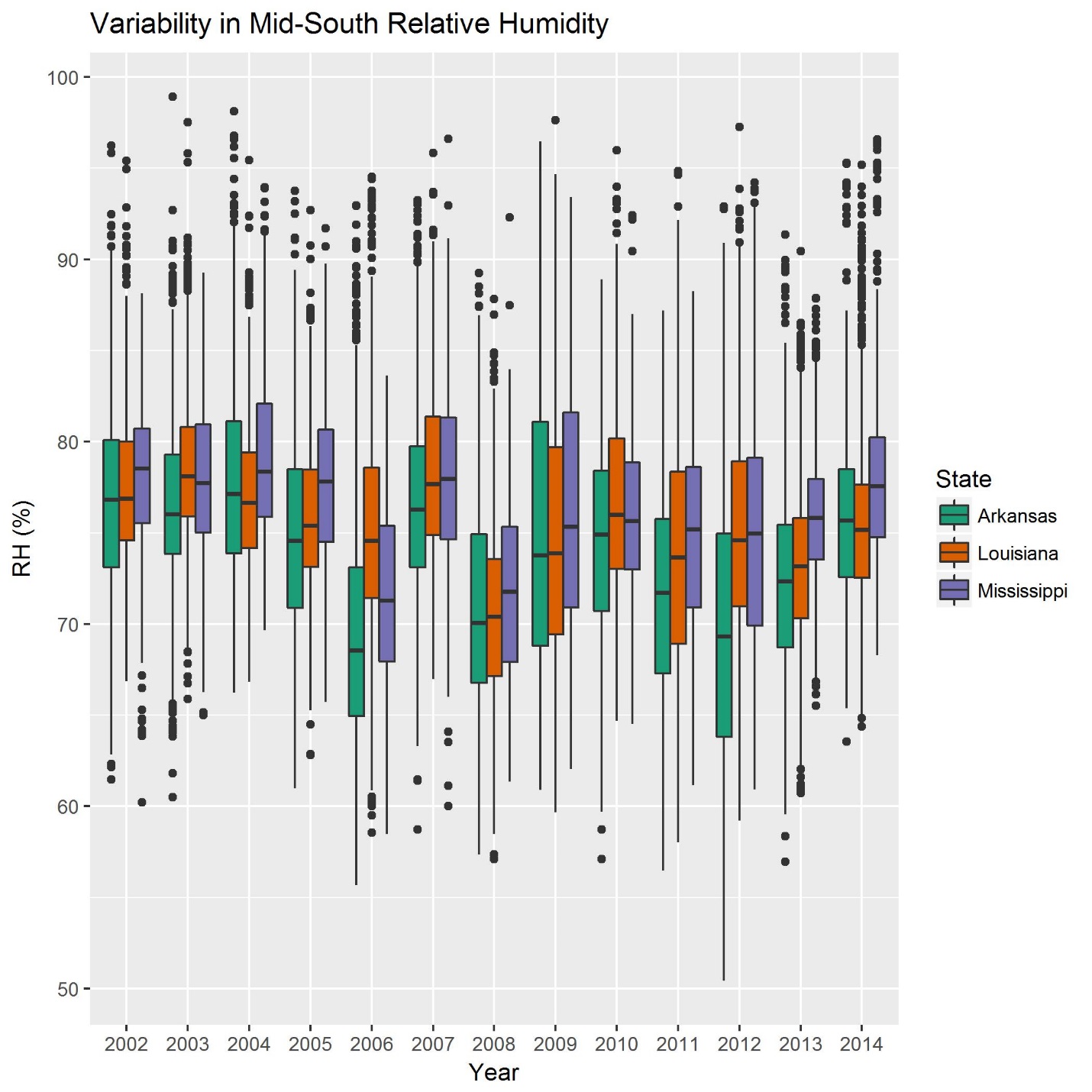
**

Supplement: S2 Fig — (DOCX) [file pone.0219199.s002.docx]

**
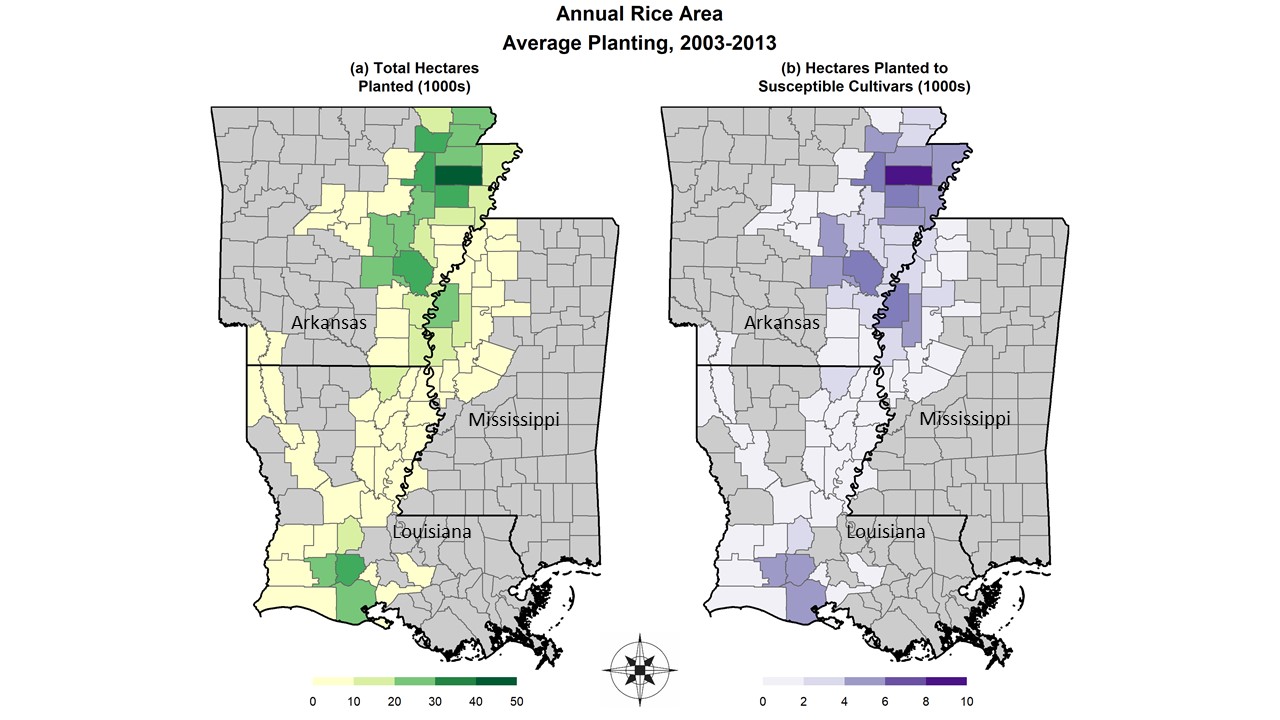
**

Supplement: S3 Fig — (DOCX) [file pone.0219199.s003.docx]

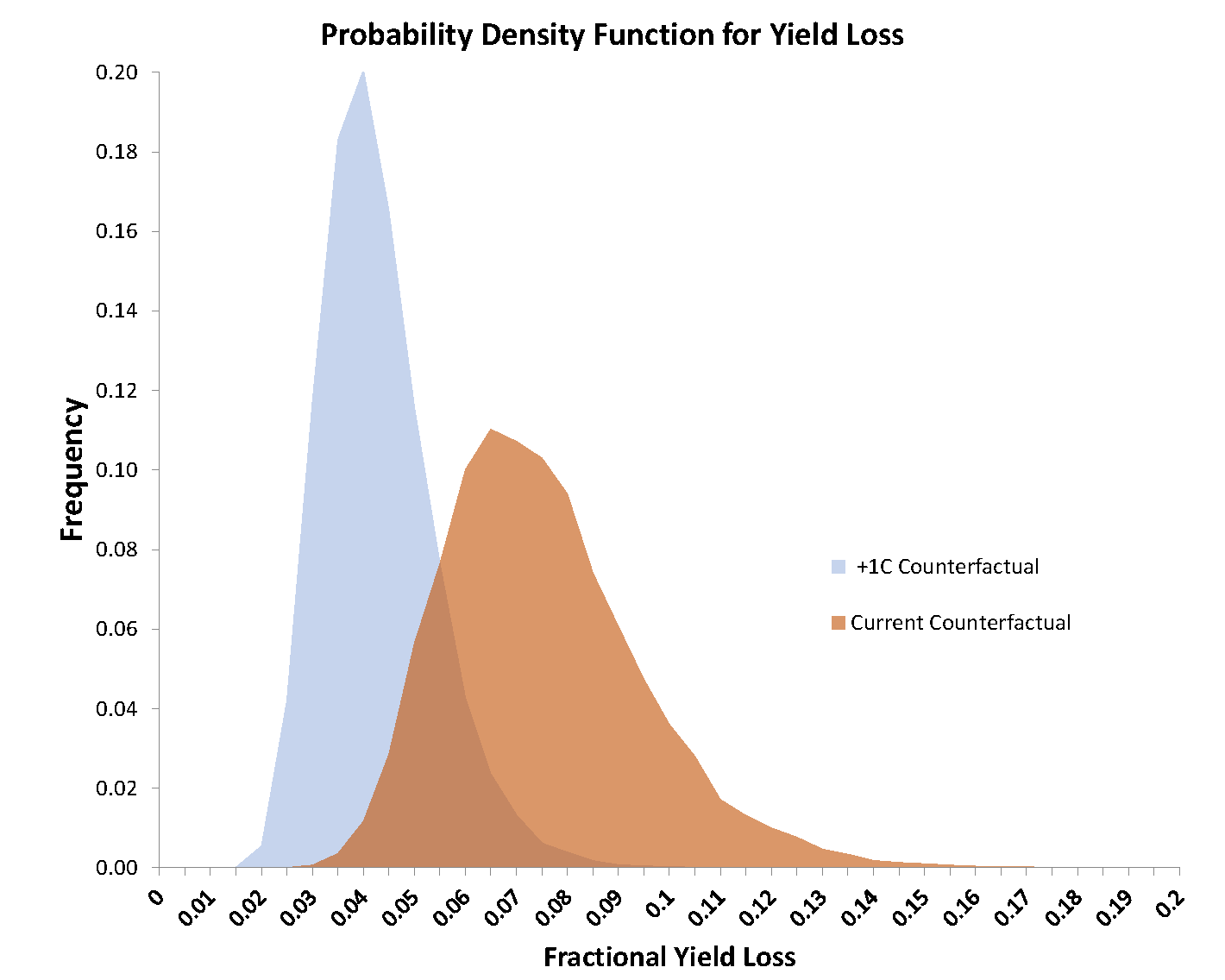

Supplement: S5 Fig — (DOCX) [file pone.0219199.s005.docx]
