# Supplementary material for: Warming increases Bacterial Panicle Blight (Burkholderia glumae) occurrences and impacts on USA rice production
Source: PLoS One. 2019 Jul 11;14(7):e0219199. doi: 10.1371/journal.pone.0219199 (PMC6623956; doi:10.1371/journal.pone.0219199)
Supplement: S4 Fig — (DOCX) [file pone.0219199.s004.docx]

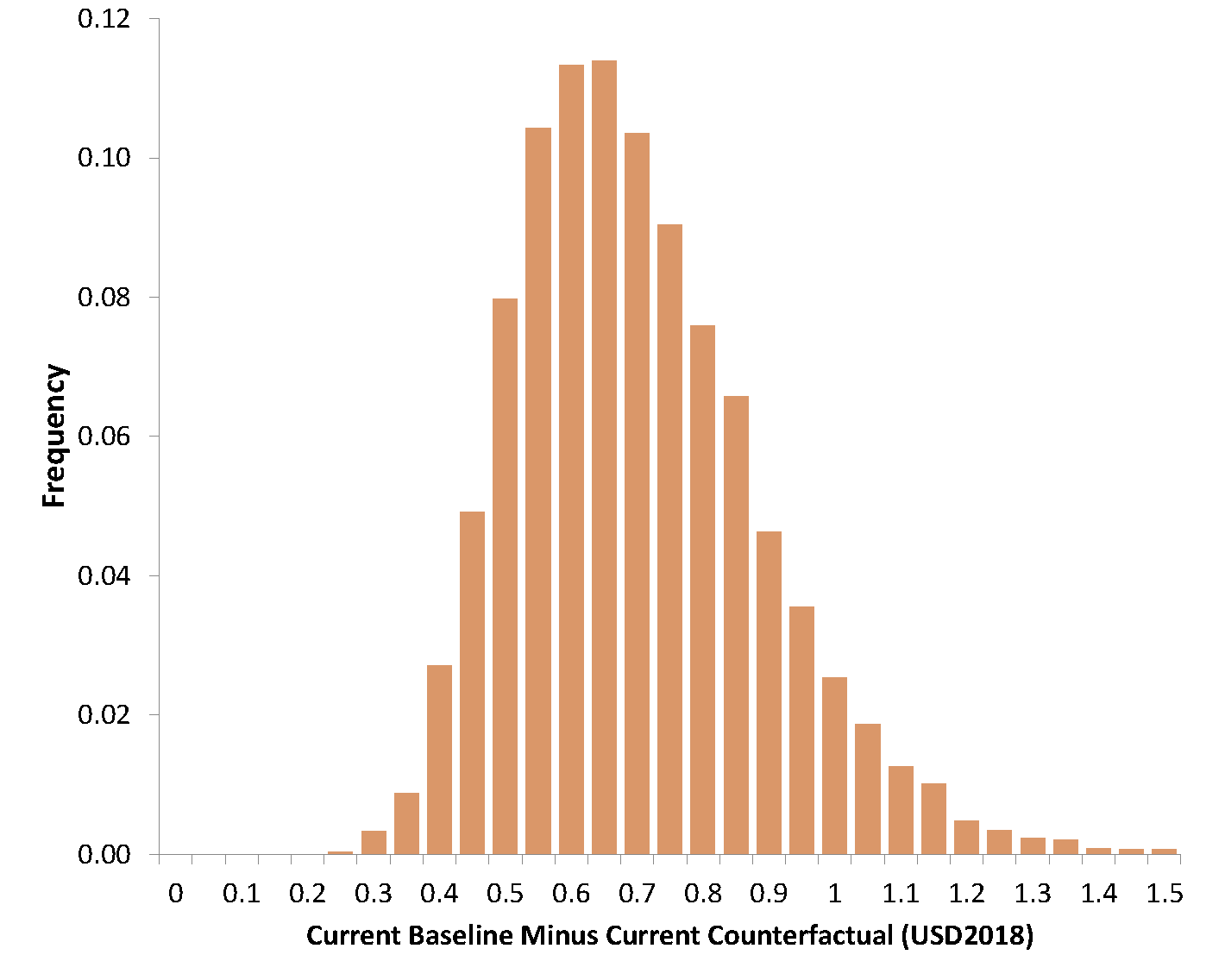


This figure presents the difference in a calculated single score (2018 USD) for the current baseline and current counterfactual scenario for 10,000 MCS runs to provide a smooth distribution.
