# Supplementary material for: Warming increases Bacterial Panicle Blight (Burkholderia glumae) occurrences and impacts on USA rice production
Source: PLoS One. 2019 Jul 11;14(7):e0219199. doi: 10.1371/journal.pone.0219199 (PMC6623956; doi:10.1371/journal.pone.0219199)
Supplement: S1 Table — (DOCX) [file pone.0219199.s006.docx]

**S1. Table. Cultivar bacterial panicle blight rating, hectares, and yield by state.**

| Genotype Name | Rice Type | Type of Genotype | Number of observation | BPB  Susceptibility |  | Highest State % of rice hectares* | | |  | Yield (Mg.ha^-1^) | | |
| --- | --- | --- | --- | --- | --- | --- | --- | --- | --- | --- | --- | --- |
|  |  |  |  |  |  | AR | LA | MS |  | AR^a^ | LA^b^ | MS^c^ |
| Ahrent | LG | Inbred | 46 | VS |  | 2.55 | - | - |  | 8.44 | - | - |
| Banks | LG | Inbred | 23 | S |  | 3.27 | - | - |  | 10.58 | - | - |
| Bengal | MG | Inbred | 252 | VS |  | 11.75 | 4.00 | - |  | 9.05 | 7.57 | - |
| Bowman | LG | Inbred | 7 | MS |  | - | - | 2.43 |  | - | - | 9.94 |
| Caffey | MG | Inbred | 8 | S |  | - | 1.01 | - |  | - | 9.19 | - |
| Catahoula | LG | Inbred | 46 | MR |  | - | 5.08 | - |  | - | 7.31 | - |
| Cheniere | LG | Inbred | 385 | MS |  | 11.54 | 24.35 | 18.26 |  | 8.79 | 7.84 | 10.41 |
| CL111 | LG | Inbred | 245 | MS |  | 6.66 | 33.78 | 12.45 |  | 8.76 | 8.29 | 11.90 |
| CL121 | LG | Inbred | 47 | VS |  | 1.39 | 2.23 | - |  | 8.01 | 6.32 | - |
| CL131 | LG | Inbred | 144 | MS |  | 14.17 | 23.26 | 19.93 |  | 8.82 | 7.64 | 9.02 |
| CL141 | LG | Inbred | 4 | VS |  | - | 0.14 | - |  | - | 5.36 | - |
| CL142 | LG | Inbred | 30 | VS |  | 8.53 | 0.14 | - |  | 7.87 | 7.87 | - |
| CL151 | LG | Inbred | 348 | MR |  | 24.57 | 34.13 | 27.77 |  | 9.18 | 9.03 | 11.89 |
| CL152 | LG | Inbred | 130 | S |  | 8.63 | 3.92 | 17.06 |  | 8.53 | 8.29 | 11.57 |
| CL161 | LG | Inbred | 373 | S |  | 20.43 | 31.46 | 18.47 |  | 8.80 | 7.72 | 8.27 |
| CL171 | LG | Inbred | 86 | S |  | 20.10 | 12.19 | 20.56 |  | 7.91 | 7.56 | 8.88 |
| CL261 | MG | Inbred | 58 | VS |  | 6.52 | 3.59 | - |  | 8.52 | 7.55 | - |
| CL271 | MG | Inbred | 4 | MS |  | - | 0.15 | - |  | - | 9.17 | - |
| CLXL4534 | LG | Hybrid | 18 | MR |  | - | - | 8.10 |  | - | - | 14.81 |
| CLXL710 | LG | Hybrid | 6 | MR |  | - | 0.15 | - |  | - | 9.40 | - |
| CLXL723 | LG | Hybrid | 175 | MR |  | 11.15 | 2.69 | 7.21 |  | 10.29 | 11.47 | 12.07 |
| CLXL729 | LG | Hybrid | 329 | MR |  | 17.19 | 8.79 | 14.42 |  | 9.94 | 10.81 | 12.33 |
| CLXL730 | LG | Hybrid | 81 | MR |  | 5.25 | 3.23 | 0.73 |  | 10.55 | 10.48 | 10.35 |
| CLXL745 | LG | Hybrid | 359 | MR |  | 33.41 | 15.75 | 22.67 |  | 8.64 | 10.59 | 12.80 |
| CLXL753 | LG | Hybrid | 111 | MR |  | 12.84 | 5.83 | 4.50 |  | 12.18 | 11.07 | 14.26 |
| CLXL8 | LG | Hybrid | 148 | R |  | 5.97 | 1.86 | 2.93 |  | 10.43 | 9.05 | 9.41 |
| Cocodrie | LG | Inbred | 648 | S |  | 30.59 | 58.05 | 78.17 |  | 8.97 | 7.71 | 10.36 |
| Cypress | LG | Inbred | 169 | S |  | 4.09 | 31.97 | 2.12 |  | 7.61 | 7.28 | 7.28 |
| Drew | LG | Inbred | 23 | MR |  | 4.61 | - | 0.08 |  | 9.09 | - | 9.09 |
| Earl | LG | Inbred | 2 | MR |  | - | 0.09 | - |  | - | 7.31 | - |
| Francis | LG | Inbred | 217 | MS |  | 17.26 | 0.91 | 2.06 |  | 9.72 | 7.28 | 10.11 |
| Jazzman2 | LG | Inbred | 15 | VS |  | - | 8.94 | - |  | - | 6.41 | - |
| Jefferson | LG | Inbred | 27 | VS |  | - | 1.35 | 1.93 |  | - | 6.42 | 6.42 |
| Jupiter | MG | Inbred | 291 | MS |  | 15.25 | 14.19 | - |  | 10.09 | 8.53 | - |
| Lagrue | LG | Inbred | 29 | VS |  | 3.57 | - | - |  | 10.02 | - | - |
| Lemont | LG | Inbred | 11 | S |  | - | - | 5.88 |  | - | - | 7.83 |
| Maybelle | LG | Inbred | 9 | S |  | - | 0.79 | - |  | - | 5.07 | - |
| Mermentau | LG | Inbred | 44 | MS |  | 5.30 | 4.30 | - |  | 9.44 | 8.55 | - |
| Neptune | MG | Inbred | 23 | VS |  | - | 1.64 | - |  | - | 7.22 | - |
| Pirogue | LG | Inbred | 13 | MS |  | - | 0.29 | - |  | - | 6.68 | - |
| Priscilla | LG | Inbred | 40 | S |  | - | - | 16.67 |  | - | - | 9.36 |
| Rex | LG | Inbred | 50 | S |  | - | - | 17.18 |  | - | - | 11.78 |
| Roy J | LG | Inbred | 75 | S |  | 15.47 | - | - |  | 10.86 | - | - |
| Saber | LG | Inbred | 6 | VS |  | - | 0.45 | 0.10 |  | - | 6.73 | 8.02 |
| Sabine | LG | Inbred | 62 | S |  | - | - | 3.23 |  | - | - | 10.39 |
| Trenasse | LG | Inbred | 43 | S |  | - | 10.40 | - |  | - | 8.20 | - |
| Wells | LG | Inbred | 473 | MS |  | 47.51 | 7.76 | 6.06 |  | 9.51 | 7.92 | 9.63 |

*Calculated by authors using annual data retrieved from the Proceedings of the Rice Technical Working Group [44].

^a^Locations in AR; Arkansas, Ashley, Chicot, Clay, Conway, Craighead, Crittenden, Cross, Desha, Drew, Faulkner, Greene, Independence, Jackson, Jefferson, Lafayette, Lawrence, Lee, Lincoln, Lonoke, Miller, Mississippi, Monroe, Perry, Phillips, Poinsett, Pope, Prairie, Pulaski, Randolph, St. Francis, White, and Woodruff.

^b^Locations in LA; Acadia, Allen, Avoyelles, Beauregard, Bossier, Caddo, Calcasieu, Caldwell, Cameron, Catahoula, Concordia, East Carroll, Evangeline, Franklin, Grant, Iberia, Iberville, Jefferson Davis, La Salle, Lafayette, Madison, Morehouse, Natchitoches, Ouachita, Point Coupee, Rapides, Red River, Richland, St Mary, St. Landry, St. Martin, Tensas, Vermilion, West Baton Rouge, and West Carroll.

^c^Locations in MS; Adams, Bolivar, Coahoma, Desoto, Grenada, Holmes, Humphreys, Issaquena, Leflore, Panola, Quitman, Sharkey, Sunflower, Tallahatchie, Tate, Tunica, Washington, and Yazoo.
