# Supplementary material for: Warming increases Bacterial Panicle Blight (Burkholderia glumae) occurrences and impacts on USA rice production
Source: PLoS One. 2019 Jul 11;14(7):e0219199. doi: 10.1371/journal.pone.0219199 (PMC6623956; doi:10.1371/journal.pone.0219199)
Supplement: S2 Table — (DOCX) [file pone.0219199.s007.docx]

S2. Table. Average Emergence and Heading Day-of-Year by State, 2003-2013

|  |  |  |  |  |  |  |  |
| --- | --- | --- | --- | --- | --- | --- | --- |
| Year | State | Average  Emergence | Minimum  Emergence | Maximum  Emergence | Average  Heading | Minimum Heading | Maximum Heading |
| 2003 | Arkansas | 125 | 119 | 131 | 213 | 207 | 219 |
| 2003 | Louisiana | 102 | 84 | 119 | 190 | 172 | 207 |
| 2003 | Mississippi | 120 | 112 | 129 | 208 | 200 | 217 |
| 2004 | Arkansas | 123 | 69 | 177 | 211 | 157 | 265 |
| 2004 | Louisiana | 94 | 85 | 102 | 182 | 173 | 190 |
| 2004 | Mississippi | 123 | 109 | 137 | 211 | 197 | 225 |
| 2005 | Arkansas | 126 | 115 | 136 | 214 | 203 | 224 |
| 2005 | Louisiana | 89 | 64 | 114 | 177 | 152 | 202 |
| 2005 | Mississippi | 119 | 94 | 144 | 207 | 182 | 232 |
| 2006 | Arkansas | 123 | 74 | 171 | 211 | 162 | 259 |
| 2006 | Louisiana | 95 | 67 | 124 | 183 | 155 | 212 |
| 2006 | Mississippi | 121 | 91 | 152 | 209 | 179 | 240 |
| 2007 | Arkansas | 122 | 109 | 136 | 210 | 197 | 224 |
| 2007 | Louisiana | 101 | 55 | 146 | 189 | 143 | 234 |
| 2007 | Mississippi | 117 | 107 | 127 | 205 | 195 | 215 |
| 2008 | Arkansas | 143 | 116 | 171 | 231 | 204 | 259 |
| 2008 | Louisiana | 95 | 77 | 112 | 183 | 165 | 200 |
| 2008 | Mississippi | 119 | 89 | 150 | 207 | 177 | 238 |
| 2009 | Arkansas | 131 | 109 | 152 | 219 | 197 | 240 |
| 2009 | Louisiana | 96 | 29 | 163 | 184 | 117 | 251 |
| 2009 | Mississippi | 124 | 94 | 155 | 212 | 182 | 243 |
| 2010 | Arkansas | 124 | 82 | 165 | 212 | 170 | 253 |
| 2010 | Louisiana | 113 | 98 | 128 | 201 | 186 | 216 |
| 2010 | Mississippi | 113 | 96 | 130 | 201 | 184 | 218 |
| 2011 | Arkansas | 133 | 133 | 133 | 221 | 221 | 221 |
| 2011 | Louisiana | 95 | 86 | 103 | 183 | 174 | 191 |
| 2011 | Mississippi | 118 | 87 | 148 | 206 | 175 | 236 |
| 2012 | Arkansas | 118 | 103 | 134 | 206 | 191 | 222 |
| 2012 | Louisiana | 101 | 42 | 160 | 189 | 130 | 248 |
| 2012 | Mississippi | 108 | 72 | 145 | 196 | 160 | 233 |
| 2013 | Arkansas | 150 | 122 | 178 | 238 | 210 | 266 |
| 2013 | Louisiana | 88 | 86 | 89 | 176 | 174 | 177 |
| 2013 | Mississippi | 123 | 97 | 150 | 211 | 185 | 238 |
