# Supplementary material for: Warming increases Bacterial Panicle Blight (Burkholderia glumae) occurrences and impacts on USA rice production
Source: PLoS One. 2019 Jul 11;14(7):e0219199. doi: 10.1371/journal.pone.0219199 (PMC6623956; doi:10.1371/journal.pone.0219199)
Supplement: S3 Table — (DOCX) [file pone.0219199.s008.docx]

|  | Susceptibility Rating | | | |
| --- | --- | --- | --- | --- |
| Year | MR | MS | S | VS |
|  |  |  |  |  |
| 2003 | - | 39,284 | 720,085 | 2,862 |
| 2004 | - | 92,908 | 855,686 | 13,513 |
| 2005 | - | 236,277 | 598,554 | - |
| 2006 | 75,234 | 56,033 | 538,934 | - |
| 2007 | 76,855 | 56,878 | 594,660 | - |
| 2008 | 1,412 | 14,580 | 753,805 | - |
| 2009 | 413,097 | 52,602 | 394,083 | 192,083 |
| 2010 | 681,629 | 116,727 | 447,991 | 106,899 |
| 2011 | 550,763 | 105,420 | 137,556 | - |
| 2012 | 219,923 | 73,441 | 230,630 | - |
| 2013 | 244,202 | 67,869 | 220,114 | - |
|  |  |  |  |  |
| Total | 2,263,115 | 912,019 | 5,492,098 | 315,357 |
