# Supplementary material for: Warming increases Bacterial Panicle Blight (Burkholderia glumae) occurrences and impacts on USA rice production
Source: PLoS One. 2019 Jul 11;14(7):e0219199. doi: 10.1371/journal.pone.0219199 (PMC6623956; doi:10.1371/journal.pone.0219199)
Supplement: S4 Table — (DOCX) [file pone.0219199.s009.docx]

| Category | Units | Description |
| --- | --- | --- |
| Ozone depletion | kg CFC-11-eq | Accumulated ozone-depleting compounds emissions |
| Global warming potential | kg CO_2_-eq | Accumulated greenhouse gas emissions  (IPCC 2006 characterization factors) |
| Acidification | m^2^ UES | Terrestrial acidification driven by acid gases;  UES =Unprotected Ecosystem |
| Eutrophication | kg NO_3_-eq | Freshwater and marine eutrophication driven by nutrient runoff |
| Carcinogens | kg C_2_H_3_Cl-eq | Human toxicity from carcinogens (e.g. pesticides, chemicals) |
| Noncarcinogens | kg C_2_H_3_Cl-eq | Human toxicity from non-carcinogens (e.g. heavy metals) |
| Fossil fuel depletion | MJ primary | Nonrenewable energy consumption |
| Eco-toxicity | kg TEG-eq w | Ecosystems toxicity units |
| Respiratory effects | kg PM_2.5_ eq | Primary and secondary particulate emissions |
| Respiratory organics | pers*ppm*hr | Human health effects from volatile organic compounds |
| Eutrophication, terrestrial | m^2^ UES | Excess nutrients on land |
| Photochemical ozone, vegetation | m^2^*ppm*hr | Damage to vegetation estimated from ozone emission |
| Ecotoxicity, terrestrial | kg TEG-eq | Toxicity associated with emissions to land |
| Nature occupation | m^2^-years agri | Agricultural land occupation – a proxy for effects to biodiversity |
|  |  |  |
