# Supplementary material for: Warming increases Bacterial Panicle Blight (Burkholderia glumae) occurrences and impacts on USA rice production
Source: PLoS One. 2019 Jul 11;14(7):e0219199. doi: 10.1371/journal.pone.0219199 (PMC6623956; doi:10.1371/journal.pone.0219199)
Supplement: S5 Table — (DOCX) [file pone.0219199.s010.docx]

| **Arkansas** | | **Mississippi** | | **Louisiana** | |
| --- | --- | --- | --- | --- | --- |
| **County** | **Outbreak Year(s)** | **County** | **Outbreak**  **Year(s)** | **Parish** | **Outbreak**  **Year(s)** |
| Arkansas | 2012 | Adams | 2003, 2005, 2007 | Acadia | 2003, 2004, 2010, 2011 |
| Chicot | 2012 | Bolivar | 2003, 2005, 2010, 2013 | Allen | 2003, 2004, 2010, 2011 |
| Clay | 2005, 2010 | Coahoma | 2013 | Avoyelles | 2003, 2004, 2010 |
| Crittenden | 2005, 2010 | Desoto | 2005 | Beauregard | 2003, 2010, 2011 |
| Cross | 2005 | Grenada | 2010 | Bossier | 2010 |
| Desha | 2012 | Holmes | 2010 | Caddo | 2010 |
| Drew | 2012 | Humphreys | 2003, 2005, 2010, 2013 | Calcasieu | 2003, 2004, 2006, 2007 |
| Greene | 2005, 2010, 2012 | Issaquena | 2003, 2005, 2010, 2013 | Caldwell | 2010 |
| Jackson | 2010, 2012 | Leflore | 2003, 2005, 2010, 2013 | Cameron | 2003, 2004, 2006, 2007, 2010, 2013 |
| Jefferson | 2012 | Quitman | 2003, 2010, 2013 | Catahoula | 2004 |
| Lawrence | 2005, 2010, 2012 | Sharkey | 2003, 2005, 2010 | Concordia | 2003, 2004 |
| Lee | 2012 | Sunflower | 2003, 2005, 2010, 2013 | East Carroll | 2003, 2004, 2010 |
| Lincoln | 2012 | Tallahatchie | 2010, 2013 | Evangeline | 2004, 2010, 2011 |
| Lonoke | 2005 | Tate | 2003, 2010 | Franklin | 2003, 2004, 2010 |
| Mississippi | 2012 | Tunica | 2003, 2005, 2013 | Grant | 2004, 2010 |
| Monroe | 2012 | Washington | 2003, 2005, 2010 | Iberia | 2003, 2004, 2010 |
| Phillips | 2012 | Yazoo | 2010 | Iberville | 2003, 2004, 2010, 2011 |
| Poinsett | 2005, 2010, 2012 |  |  | Jefferson Davis | 2003, 2004, 2005, 2006, 2007, 2010, 2011 |
| Prairie | 2012 |  |  | La Salle | 2004, 2010 |
| Randolph | 2005 |  |  | Lafayette | 2003, 2004, 2010 |
| Woodruff | 2012 |  |  | Madison | 2004 |
|  |  |  |  | Morehouse | 2010 |
|  |  |  |  | Natchitoches | 2010, 2011 |
|  |  |  |  | Rapides | 2004, 2010 |
|  |  |  |  | Red River | 2010 |
|  |  |  |  | Richland | 2004, 2010 |
|  |  |  |  | Tensas | 2003, 2004 |
|  |  |  |  | Vermilion | 2004, 2005, 2007, 2010, 2011 |
|  |  |  |  | West Baton Rouge | 2003, 2004, 2010 |
|  |  |  |  | West Carroll | 2004, 2010 |
