# Supplementary material for: Warming increases Bacterial Panicle Blight (Burkholderia glumae) occurrences and impacts on USA rice production
Source: PLoS One. 2019 Jul 11;14(7):e0219199. doi: 10.1371/journal.pone.0219199 (PMC6623956; doi:10.1371/journal.pone.0219199)
Supplement: S6 Table — (DOCX) [file pone.0219199.s011.docx]

|  | **Arkansas** | | | **Louisiana** | | | **Total LMDR** | | |
| --- | --- | --- | --- | --- | --- | --- | --- | --- | --- |
| Year | Baseline Production  (MT) | Production Loss  (MT) | % Loss | Baseline Production  (MT) | Production Loss  (MT) | % Loss | Baseline Production  (MT) | Production Loss  (MT) | % Loss |
|  |  |  |  |  |  |  |  |  |  |
| 2003 | 628,916 | 0 | 0.00 | 50,850 | 9,026 | 15.07 | 679,766 | 9,026 | 1.31 |
| 2004 | 477,528 | 0 | 0.00 | 51,016 | 6,442 | 11.21 | 528,544 | 6,442 | 1.20 |
| 2005 | 316,790 | 33,588 | 9.59 | 26,642 | 1,345 | 4.81 | 343,431 | 34,934 | 9.23 |
| 2006 | 322,567 | 0 | 0.00 | 46,620 | 332 | 0.71 | 369,187 | 332 | 0.09 |
| 2007 | 619,723 | 0 | 0.00 | 66,419 | 3,427 | 4.91 | 686,142 | 3,427 | 0.50 |
| 2008 | 352,611 | 0 | 0.00 | 38,730 | 0 | 0.00 | 391,342 | 0 | 0.00 |
| 2009 | 771,248 | 0 | 0.00 | 184,223 | 0 | 0.00 | 955,471 | 0 | 0.00 |
| 2010 | 668,867 | 54,316 | 7.51 | 91,058 | 5,808 | 6.00 | 759,924 | 60,124 | 7.33 |
| 2011 | 802,120 | 0 | 0.00 | 141,155 | 8,297 | 5.55 | 943,275 | 8,297 | 0.87 |
| 2012 | 441,667 | 21,912 | 4.73 | 79,763 | 0 | 0.00 | 521,430 | 21,912 | 4.03 |
| 2013 | 405,973 | 0 | 0.00 | 57,251 | 0 | 0.00 | 463,224 | 0 | 0.00 |
|  |  |  | 0 |  |  |  |  |  |  |
| **Total** | **5,808,010** | **109,817** | **1.86** | **833,727** | **34,676** | **3.99** | **6,641,737** | **144,493** | **2.13** |
