# Supplementary material for: Warming increases Bacterial Panicle Blight (Burkholderia glumae) occurrences and impacts on USA rice production
Source: PLoS One. 2019 Jul 11;14(7):e0219199. doi: 10.1371/journal.pone.0219199 (PMC6623956; doi:10.1371/journal.pone.0219199)
Supplement: S7 Table — (DOCX) [file pone.0219199.s012.docx]

|  | **Arkansas** | | | **Louisiana** | | | **Mississippi** | | | **Total LMDR** | | |
| --- | --- | --- | --- | --- | --- | --- | --- | --- | --- | --- | --- | --- |
| Year | Baseline Production  (MT) | Production Loss  (MT) | % Loss | Baseline Production  (MT) | Production Loss  (MT) | % Loss | Baseline Production  (MT) | Production Loss  (MT) | % Loss | Baseline Production  (MT) | Production Loss  (MT) | % Loss |
|  |  |  |  |  |  |  |  |  |  |  |  |  |
| 2003 | 4,939,145 | 0 | 0.00 | 1,288,820 | 99,584 | 7.17 | 762,230 | 74,489 | 8.90 | 6,990,196 | 174,073 | 2.43 |
| 2004 | 4,295,983 | 0 | 0.00 | 1,465,251 | 112,970 | 7.16 | 962,108 | 0 | 0.00 | 6,723,342 | 112,970 | 1.65 |
| 2005 | 5,957,018 | 159,487 | 2.61 | 1,596,245 | 37,653 | 2.30 | 834,831 | 56,231 | 6.31 | 8,388,094 | 253,371 | 2.93 |
| 2006 | 4,389,534 | 0 | 0.00 | 1,144,209 | 24,174 | 2.07 | 670,201 | 0 | 0.00 | 6,203,944 | 24,174 | 0.39 |
| 2007 | 4,183,783 | 0 | 0.00 | 985,772 | 29,330 | 2.89 | 728,393 | 0 | 0.00 | 5,897,948 | 29,330 | 0.49 |
| 2008 | 2,861,253 | 0 | 0.00 | 1,079,690 | 0 | 0.00 | 769,797 | 0 | 0.00 | 4,710,740 | 0 | 0.00 |
| 2009 | 3,477,989 | 0 | 0.00 | 1,326,152 | 0 | 0.00 | 1,051,864 | 0 | 0.00 | 5,856,006 | 0 | 0.00 |
| 2010 | 5,565,979 | 104,931 | 1.85 | 1,498,495 | 74,884 | 4.76 | 1,353,247 | 97,828 | 6.74 | 8,417,721 | 277,643 | 3.19 |
| 2011 | 2,624,339 | 0 | 0.00 | 1,223,206 | 46,549 | 3.67 | 793,739 | 0 | 0.00 | 4,641,284 | 46,549 | 0.99 |
| 2012 | 3,909,378 | 164,014 | 4.03 | 1,101,172 | 0 | 0.00 | 523,995 | 0 | 0.00 | 5,534,545 | 164,014 | 2.88 |
| 2013 | 3,196,595 | 0 | 0.00 | 1,239,316 | 2,046 | 0.16 | 532,185 | 35,540 | 6.26 | 4,968,097 | 37,586 | 0.75 |
|  |  |  |  |  |  |  |  |  |  |  |  |  |
| **Total** | **45,400,998** | **428,432** | **0.93** | **13,948,328** | **427,190** | **2.97** | **8,982,591** | **264,089** | **2.86** | **68,331,917** | **1,119,710** | **1.61** |
