# Supplementary material for: Warming increases Bacterial Panicle Blight (Burkholderia glumae) occurrences and impacts on USA rice production
Source: PLoS One. 2019 Jul 11;14(7):e0219199. doi: 10.1371/journal.pone.0219199 (PMC6623956; doi:10.1371/journal.pone.0219199)
Supplement: S8 Table — The Values Represent the Percent of Cumulative Annual (EU) Impact in Each Category Attributable to Rice Consumption. (DOCX) [file pone.0219199.s013.docx]

| Impact category | Baseline | Panicle Resistant | Panicle Resistant  plus 1^o^C |
| --- | --- | --- | --- |
| Ecotoxicity, terrestrial | 8.16% | 8.05% | 7.95% |
| Ecotoxicity, aquatic | 3.01% | 3.00% | 2.99% |
| Respiratory organics | 2.07% | 2.04% | 2.02% |
| Photochemical ozone, vegetat. | 1.48% | 1.45% | 1.43% |
| Eutrophication, terrestrial | 1.43% | 1.41% | 1.39% |
| Human toxicity, carcinogens | 1.35% | 1.32% | 1.29% |
| Respiratory inorganics | 1.09% | 1.07% | 1.05% |
| Global warming, fossil | 1.06% | 1.05% | 1.04% |
| Mineral extraction | 0.98% | 0.96% | 0.94% |
| Eutrophication, aquatic | 0.81% | 0.80% | 0.80% |
| Non-renewable energy | 0.56% | 0.55% | 0.54% |
| Human toxicity, non-carc. | 0.43% | 0.38% | 0.35% |
| Acidification | 0.34% | 0.34% | 0.33% |
| Nature occupation | 0.04% | 0.04% | 0.04% |
