# Supplementary material for: Warming increases Bacterial Panicle Blight (Burkholderia glumae) occurrences and impacts on USA rice production
Source: PLoS One. 2019 Jul 11;14(7):e0219199. doi: 10.1371/journal.pone.0219199 (PMC6623956; doi:10.1371/journal.pone.0219199)
Supplement: S1 Appendix — (DOCX) [file pone.0219199.s014.docx]

**Supplementary Information:**

**Appendix A | Description of the partial spatial equilibrium model of the global rice economy.**

We use a partial, spatial equilibrium model of the global rice economy to assess the economic impact of BPB. The model simulates the behavior of the entire rice supply chain, from input markets all the way up to the aggregate final demand, in multiple countries/regions (set $R$) around the world.

Production of endogenous rice commodities (set $CE$) is specified as a weak-separable, constant return to scale production function:

$$Y_{c,r}=H_{c,r}\left\{ G_{c,r}\left( {FAC}_{c,r} \right),{INT}_{c,r} \right\} \forall c\in CE, r\in R, (1)$$

Where $Y$ represents output, $H$ and $G$ are technology functional forms, $FAC$ is the set of factors of production, and $INT$ is the set of intermediate inputs.

Defining $G$ in $(1)$ as a constant elasticity of substitution (CES) function, the derived demand for factor of production, $QFC$, is

${QFC}_{f,c,r}*{AFC}_{f,c,r}={QVA}_{c,r}*{SVA}_{f,c,r}*\left[ \frac{{PFC}_{f,c,r}}{{PVA}_{c,r}*{AFC}_{f,c,r}} \right]^{{-\sigma VA}_{c,r}} \forall f\in FAC, c\in CE, r\in R, (2)$

$${PVA}_{c,r}=\left[ \sum_{f} {SVA}_{f,c,r}*\left( \frac{{PFC}_{f,c,r}}{{AFC}_{f,c,r}} \right)^{1{-\sigma VA}_{c,r}} \right]^{\frac{1}{1{-\sigma VA}_{c,r}}} \forall c\in CE, r\in R, (3)$$

Where $AFC$, $PFC$, and $SVA$ are a factor-, sector-, and region-specific augmenting technical change variable, factor price variable, and cost share in value added, respectively, and $QVA$ and $PVA$ are a sector- and region-specific derived demand and price for the value added composite, respectively. Finally, $\sigma VA$ is the sector- and region-specific elasticity of substitution in value added.

Defining $H$ in $(1)$ as a constant elasticity of substitution (CES) function, the derived demands for intermediate inputs $QIC$, and for the composite value added ${QVA}_{c,r}$, are

${QIC}_{i,c,r}*{AIC}_{i,c,r}=\frac{Y_{c,r}}{{AY}_{c,r}}*{SITC}_{i,c,r}*\left[ \frac{{PIC}_{i,c,r}}{{PY}_{c,r}}*{AIC}_{f,c,r}*{AY}_{c,r} \right]^{{-\sigma Y}_{c,r}}, \forall i\in INT, c\in CE, r\in R, (4)$

${QVA}_{c,r}*{AVA}_{c,r}=\frac{Y_{c,r}}{{AY}_{c,r}}*{SVATC}_{c,r}*\left[ \frac{{PVA}_{c,r}}{{PY}_{c,r}}*{AVA}_{c,r}*{AY}_{c,r} \right]^{{-\sigma Y}_{c,r}}, \forall c\in CE, r\in R, (5)$

Where $AIC$, $PIC$, and $SITC$ are input-, sector-, and region-specific input augmenting technical change variable, input price variable, and input cost share in total cost, respectively. Furthermore, $AVA$, $AY$, and $PY$, and $SVATC$ are sector- and region-specific value-added augmenting technical change variable, output augmenting technical change variable, output price variable, and value-added cost share in total cost, respectively. Finally, $\sigma Y$ is the sector- and region-specific elasticity of substitution in final output.

The model assumes zero profits in production (Equation (6)) and equilibrium in output markets, Equation (7i) for paddy rice commodities^[[1]](#footnote-4)^, and (7ii) for other rice commodities^[[2]](#footnote-5)^).

$${PY}_{c,r}=\frac{\left[ {SVATC}_{c,r}*\left( \frac{{PVA}_{c,r}}{{AVA}_{c,r}} \right)^{1{-\sigma Y}_{c,r}}+\sum_{i} {SITC}_{i,c,r}*\left( \frac{{PIC}_{i,c,r}}{{AIC}_{i,c,r}} \right)^{1{-\sigma Y}_{c,r}} \right]^{\frac{1}{1{-\sigma Y}_{c,r}}}}{{AY}_{c,r}}, \forall c\in CE, r\in R, (6)$$

$$Y_{c,r}={QD}_{c,r}+\sum_{s} {QBX}_{c,r,s}+{QK}_{c,r} , \forall c\in CP, r\in R, (7i)$$

$$Y_{c,r}={QD}_{c,r}+\sum_{s} {QBX}_{c,r,s}, \forall c\in CCP, r\in R, (7ii)$$

Where $QD$ represent the volume of output $c$ sold in the domestic market, $QK$ is the change in stocks^[[3]](#footnote-6)^ of good c, and $QBX$ is the volume of bilateral exports of $c$ from region $r$ to region $s$.

Import demand follows the Armington approach (Armington, 1969), by which imports by source and domestic production are treated as heterogeneous products. Agents first decide on the sourcing of imports (Equation (8)) based on the relative level of prices from each source (Equation (9)).

${QBX}_{c,s,r}={QM}_{c,r}*{SMS}_{c,s,r}*\left[ \frac{{PMMS}_{c,s,r}}{{PMM}_{c,r}} \right]^{{-\sigma M}_{c,r}}, \forall c\in CE, r\in R, s\in R, (8)$

$${PMM}_{c,r}=\left[ \sum_{s} {SMS}_{c,s,r}*{{PMMS}_{c,s,r}}^{1{-\sigma M}_{c,r}} \right]^{\frac{1}{1{-\sigma M}_{c,r}}}, \forall c\in CE, r\in R, (9)$$

Where $PMMS$ is the market price of import good $c$ into region $r$ from source $s$, $PMM$ is the composite market price of import good $c$ in $r$, $QM$ is the demand for the composite import good $c$ in $r$, and $SMS$ is the value-share of good $c$’s import into $r$ by source $s$. ${\sigma M}_{c,r}$ is the elasticity of substitution of imported good $c$ in $r$ by source.

After sourcing imports, agents then decide on the optimal mix of imported and domestic products (Equation (10) and (11)) based on their relative price levels (Equation (12)):

${QM}_{c,r}={QQ}_{c,r}*{SMQ}_{c,r}*\left[ {{PMM}_{c,r}}/{{PQ}_{c,r}} \right]^{{-\sigma Q}_{c,r}}, \forall c\in CE, r\in R, (10)$

${QD}_{c,r}={QQ}_{c,r}*{SDQ}_{c,r}*\left[ {{PY}_{c,r}}/{{PQ}_{c,r}} \right]^{{-\sigma Q}_{c,r}} , \forall c\in CE, r\in R, (11)$

${PQ}_{c,r}=\left[ {SMQ}_{c,r}*{{PMM}_{c,r}}^{1{-\sigma Q}_{c,r}}+{SDQ}_{c,r}*{{PY}_{c,r}}^{1{-\sigma Q}_{c,r}} \right]^{\frac{1}{1{-\sigma Q}_{c,r}}} , \forall c\in CE, r\in R, (12)$

Where $PQ$ is the market price of composite good $c$ in region $r$, $QQ$ is the output of composite good $c$ in $r$, and $SMQ$ and SDQ are the value-shares of the import composite and domestic good $c$ in $r$. ${\sigma Q}_{c,r}$ is the elasticity of substitution between domestic and imported good $c$ in $r$.

Final demand for milled rice $c\in CFC$ in region $r$, is the product of population and per-capita demand $D_{c,r}$, which is specified as a double log function of income and prices (Equation (13)). $Z_{r}$ represents income by region, $\varphi_{r}$ is the income demand elasticity, and $\omega_{c,g,r}$ is the matrix of own and cross-price demand elasticities:

$\log D_{c,r}=\varphi_{r}*log Z_{r}+\sum_{g \in FC} \omega_{c,g,r}*\log{PQ}_{g,r} , \forall c\in CFC, r\in R. (13)$

The supply of exogenous intermediate inputs (seeds, fertilizers, pesticides, energy, and water), capital, and labor are specified as perfectly elastic, thus their prices ($PFC$) are treated as constant, exogenous variables. Land is considered the only factor with limited supply. Hence, sectoral output $Y$ is constrained only by the supply of land $L_{c,r}$ used in the production of paddy rice, which is represented by a double log function of land rental rates ${PL}_{c,r}$:

$\log L_{c,r}=\theta_{c,r}\log{PL}_{c,r} , \forall c\in CP, r\in R. (14)$

The land own-price supply elasticity $\theta_{c,r}$ are calibrated following Keller (1976) to reflect rice supply elasticities found in the literature.

1. Set $CP=\left\{ LGP,MGP,FRP \right\}. CP\in CE$ [↑](#footnote-ref-4)
2. Set $CCP=CE - CP=\{LGB,MGB,FRB,LGW,MGW,FRW\}$ [↑](#footnote-ref-5)
3. Only stocks of paddy rice are allowed. Thus ${QK}_{c,r}$ is defined over the commodity subset $CP$. [↑](#footnote-ref-6)
